# Medical Students’ Perception Regarding the Re-emerging Monkeypox Virus: An Institution-Based Cross-Sectional Study From Saudi Arabia

**DOI:** 10.7759/cureus.28060

**Published:** 2022-08-16

**Authors:** Najim Z Alshahrani, Sayan Mitra, Ali A Alkuwaiti, Maathir N Alhumam, Sarah Muqbil B Altmimi, Mohammad Hassan M Alamri, Zainab Atallah S Albalawi, Majed W Almorgi, Hamad Khulaif D Alharbi, Sultan M Alshahrani

**Affiliations:** 1 Family and Community Medicine, Faculty of Medicine, University of Jeddah, Jeddah, SAU; 2 Medicine and Health, University of Sydney, Sydney, AUS; 3 Medicine, King Faisal University, Alahsa, SAU; 4 Medicine, University of Tabuk, Tabuk, SAU; 5 Medicine, King Saud Bin Abdulaziz University for Health Sciences, Riyadh, SAU; 6 General Physician, Maternity and Children Hospital, Tabuk, SAU; 7 Medicine, Taif University, Taif, SAU; 8 Medicine, Al Jouf University, Al-Jouf, SAU; 9 Pharmacy, King Khalid University, Abha, SAU

**Keywords:** zoonotic disease, viruses, saudi arabia, outbreak, monkeypox, medical student, knowledge, epidemic, awareness, attitude

## Abstract

Introduction: The recent multi-nation outbreaks of human monkeypox in non-endemic areas have created an emerging public health issue. Medical students who will become future healthcare providers are directly associated with community people and can easily sensitize the general population, so it is crucial to assess their degree of knowledge and attitudes regarding recently emerging infections or pathogens. However, studies on medical students' perception of the monkeypox virus are scarce in Saudi Arabia. Therefore, the objective of this study was to assess the monkeypox virus-related knowledge and attitudes among medical students in the country.

Methods: A cross-sectional study was conducted from May to July 2022 among undergraduate medical students at King Khalid University, Abha, Saudi Arabia. A systematic random sampling technique was applied to select the study participants. A self-administered questionnaire was used to gather data on sociodemographic characteristics, knowledge and attitudes toward the monkeypox virus. Descriptive statistics and Chi-square tests were performed.

Results: A total of 314 medical students were recruited for this study. The findings from this study showed that the vast majority of medical students (72%) had poor knowledge about the monkeypox virus. Respondents’ age, grade point average (GPA), fathers’ education level, and training received about the monkeypox virus were significantly associated with the level of knowledge about the monkeypox virus (p < 0.05). Nearly half of the respondents (45.9%) agreed that the monkeypox virus could be transmitted to Saudi Arabia. Overall, this study showed that the awareness levels regarding the monkeypox virus were significantly higher among seniors as compared to junior students.

Conclusion: The study found poor knowledge of the monkeypox virus among currently enrolled medical students in the country’s highest-ranked medical school. This finding emphasizes the urgent need to increase their knowledge because controlling outbreaks requires significant cooperation from knowledgeable and skilled healthcare providers.

## Introduction

In many regions of the world, the incidence of morbidity, mortality, and epidemics has increased in recent years due to the reappearance and evolution of numerous animal-borne diseases [1]. Monkeypox is a disease caused by the zoonotic monkeypox virus, and it is one of the four recognized poxvirus species infections following the eradication of smallpox. Even though it affects various taxonomic species, the original host remains unknown [2]. In the Democratic Republic of the Congo and Sooty Mangabey, Cote d'Ivoire, the monkeypox virus was isolated from animals; a wild rope squirrel and a wild-living monkey [2,3]. The recent resurgence began with the largest outbreak of monkeypox, with over 200 confirmed cases occurring in Nigeria in 2019, according to the World Health Organization (WHO). In addition, they reported that 11 African countries and four regions outside of Africa were affected by monkeypox, including the United Kingdom, the United States of America, Singapore, and Israel [4].

The incubation period of the monkeypox virus ranges from five to 21 days [5]. The disease is typically transmitted through contact with an infected animal's skin or body fluids, such as a monkey, rat, or squirrel. Human-to-human transmission occurs primarily through contact with skin lesions, respiratory particles, and bodily fluids. Health workers and other members' close contact with personnel are more susceptible to respiratory droplet particle exposure [6]. Moreover, vertical transmission of the monkeypox virus may occur (mother to fetus) [7]. Sexual contact is another possible mode of transmission [8]. Recent research in Italy on the seminal fluid of three patients with monkeypox revealed the presence of monkeypox virus DNA in all three patients. As in the current outbreak, most cases were detected in bisexuals, men who engage in sexual activity with other men, people with multiple sexual partners, and those who engage in unprotected sex [9,10]. The blood-borne route is another mode of transmission for the monkeypox virus, which can also be transmitted from animals to humans. Consequently, hunters are at risk as they are highly exposed to infected animals, particularly during the slaughtering process. They risk accidental fecal-oral route transmission by inhaling aerosolized particles in the affected animal's fur and urine or during carcass management [11,12].

Since 1980, when smallpox was eradicated, the monkeypox virus has been one of the most pathogenic poxviruses. However, its recent resurgence posed a threat to global health with (WHO) declaring it to be a public health emergency in July 2022. Due to its alarming global cause, combined efforts should be made to raise awareness by promoting preventative measures, such as educating healthcare workers and the general public about early detection, response activities, and rapid risk assessment [13]. To achieve health security, it is necessary to recruit physicians, public health experts, veterinarians, and virologists to develop vaccines and provide other preventative and therapeutic measures [14]. People vaccinated against smallpox are 85% protected overall. Unfortunately, due to the rise in anti-vaccination campaigns, this protection is in jeopardy of being discontinued for many of the population, particularly children [15].

Saudi Arabia reported its first confirmed case of the monkeypox virus in a foreign patient on July 14, 2022 [16]. Information regarding the monkeypox virus is especially vital for medical students who are future doctors. There is a recent study done in Saudi Arabia conducted among medical practitioners about their knowledge of the monkeypox virus, its findings showed only 18.6% of them reported having some understanding of managing monkeypox [17]. Another recent study reported that knowledge of monkeypox infection was slightly poor among the Saudi population [5]. There are no studies conducted worldwide among medical students. However, there have been few studies conducted among general practitioners in Indonesia [17-19]. As the country hosts the umrah and hajj mass gatherings annually, effective management of the monkeypox virus outbreak is particularly important here. This year, approximately one million domestic and international pilgrims will attend the hajj, most of whom are from regions where the monkeypox virus is endemic. The communities' and healthcare providers' awareness of the virus is influenced by their knowledge and attitude regarding the virus [20,21]. Medical students who will become future healthcare providers are directly associated with community people and can easily sensitize the general public, so it is crucial to assess their degree of knowledge and attitudes regarding recently emerging infections or pathogens. Thus, this study aimed to evaluate the knowledge and attitudes toward the monkeypox virus among medical students at King Khalid University in Abha, Saudi Arabia.

## Materials and methods

Study setting and duration

This study was carried out among undergraduate medical students at King Khalid University, Abha, Saudi Arabia, who were enrolled in their second, third, fourth, fifth, and sixth academic years. This public university, located in southern Saudi Arabia, comprises 29 colleges with a range of academic disciplines, including medicine, engineering, and computer science. Approximately 60,000 students are enrolled at this university (as of the 2019 academic year). The southern region of the country has a growing population, and this renowned institution meets their basic needs, such as education, and helps policymakers by exploring scientific knowledge, which makes our study area suitable for conducting research on such emerging public health issues. The study period was from May 24, 2022 to July 20, 2022.

Study design, subject, and sampling

An institution-based cross-sectional study was carried out among 314 medical students to assess their knowledge and attitude about monkeypox virus. The participants were enrolled after meeting the following inclusion requirements: (i) being undergraduate medical students and (ii) Saudi citizens by birth. Students who were reluctant to participate in the study or had major illnesses or mental issues were not allowed to participate. A systematic random sampling technique was used to choose the participants, taking into account the individuals' academic year and gender. The sample size was calculated by the appliance of the single sample proportion formula [22], n = z2pq/d2, where n is the required sample size, z is 1.96 at a 95% confidence interval, d is the margin of error at 5% (standard deviation of 0.05), and q = 1-p. In this study, 50% (p = 50%) of the anticipated prevalence of students' knowledge of the monkeypox virus was used (since there were no similar investigations in Saudi Arabia). Thus, a minimum sample size of 384 students was obtained. However, a total of 314 samples were finally included in this study. We were unable to obtain the calculated sample size because of the exclusion criteria and the participants' refusal to participate.

Content of the study tool

A structured, anonymous and close-ended questionnaire was developed for this study. The electronic survey tool consisted of three parts: (i) participants’ sociodemographic information, (ii) assessment of participants' knowledge of the monkeypox virus, and (iii) assessment of attitudes toward the monkeypox virus. The first part of the questionnaire includes students’ sociodemographic information such as age, gender, education year, grade point average (GPA), fathers and mothers education level, source of information about the monkeypox virus, etc. The participants' knowledge of the monkeypox virus was assessed in the following section, which included 20 questions. The questions included information about the first isolation of the monkeypox virus, where it occurred, and which part of the world is nowadays the most affected. In addition, they were questioned about the mode of transmission, symptoms, incubation period, the most severe complications, and the likelihood of transmission of the virus to Saudi Arabia. Finally, to assess attitudes toward the monkeypox virus, a set of 10 statements on a five-point Likert scale was used (strongly disagree, disagree, neutral, agree, and strongly agree). These statements are intended to elicit respondents' perspectives on the potential to control the monkeypox virus globally and their attitudes toward learning more about it, travel medicine and re-emerging infectious diseases.

Reliability and validity of the questionnaire

To prepare the questionnaire, the authors of this study searched for and reviewed all possible previous research on “monkeypox virus epidemiology” and “monkeypox knowledge and attitudes.” The different databases including EMBASE, Cochrane Library, and PubMed, and the websites of the CDC, WHO, and Google Scholar were searched until May 25, 2022 for published research. The most relevant articles were chosen to construct the questionnaire [5,17]. The content validity of the questionnaire was examined using the translation back-translation method. The questionnaire was initially created in English and then, in order to ensure consistency and minimize bias, it was back-translated into Arabic (the local language) by a bilingual expert. Moreover, the validity of the content was evaluated by three public health researchers who are experts in the relevant fields. The questionnaire was piloted among 10 medical students to ensure that there were no unclear questions (the findings were excluded from the final analysis). The internal consistency of the questionnaire was measured using Cronbach's alpha and found to have an acceptable level of reliability (i.e., Cronbach's alpha = 0.84).

Data collection procedure

Prior to data collection, ethical clearance was obtained from the Research Ethics Committee (REC) of King Khalid University, Saudi Arabia. For data collection and entry, we used Google Forms and Excel sheets. Initially, we collected the students’ email addresses from the respective department of the institution. We emailed the questionnaire to the participants’ institutional email addresses. Each participant was briefed on the purpose and objectives of the study before being asked for participation consent. The participants were also assured that their responses to the questionnaire would be kept confidential. In addition, the participants were informed that they could withdraw from the study at any time and that participation was not required for their course.

Statistical analysis

Data were analyzed using Statistical Package for social sciences (SPSS) software (version 23, IBM Corp., Armonk, NY, USA). Descriptive statistics such as frequency and percentage were computed to summarize the variables of interest (for example, socio-demographic information, and knowledge and attitudes items). A scoring system ranging from 0 to 20 points was created to assess the level of knowledge regarding the monkeypox virus. For the knowledge item (20 questions), a “1” point was given for correct responses, and a “0” point was assigned for unknown or incorrect responses. The total score was used to determine the level of knowledge classification, with a score of 11 indicating a poor level of knowledge and 11 or higher indicating a good level of knowledge. The Pearson Chi-Square test was used to assess the associations between variables. P-values less than 0.05 were regarded as statistically significant.

## Results

Three hundred fourteen of the 400 medical students who were invited to take part in the study completed the questionnaire, yielding a response rate of 78.5% (Table 1). Male students accounted for 41.7% (n = 131) of the responses, while female students accounted for 58.3% (n = 183). The majority of participants (85%) were older than 21 years. Approximately half of the respondents had a GPA ≥ 3.75. In most cases, both parents' educational level was at or above the university level. Approximately 57.3% (n =180) of participants had a COVID-19 infection. When asked about the source of monkeypox virus-related information, many participants selected Twitter.

**Table 1:** The sociodemographic characteristics of the medical students who participated in the survey (N= 314) *Each question was asked separately      **Multiple choices allow for this question

Characteristics	N (%)
Gender	
Male	131 (41.7)
Female	183 (58.3)
Age	
≤21	47 (15)
>21	267 (85)
Educational Year	
2nd year	67 (21.3)
3rd year	48 (15.3)
4th year	64 (20.4)
5th year	65 (20.7)
6th year	70 (22.3)
GPA (out of 5)	
<2.5	92 (29.3)
2.5–3.74	94 (29.9)
≥3.75	128 (40.8)
Father's education	
< University level	76 (24.2)
≥University level	238 (75.8)
Mother's education	
< University level​​​​​​​	72 (22.9)
≥University level	242 (77.1)
Infected with COVID-19	
Yes	180 (57.3)
No	134 (42.7)
Received training programs about monkeypox	
Yes	120 (38.2)
No	194 (61.8)
Source of Information about monkeypox **	
Twitter	195 (62.1)
Snapchat	151 (48.1)
Television	97 (30.8)
WhatsApp group	51 (16.2)
Friends and relatives	13 (4.2)
Research articles	46 (14.7)

Table 2 demonstrates the medical student's knowledge of the monkeypox virus. Most participants (n = 294; 93.8%) believe that the monkeypox virus has become an epidemic worldwide and is more prevalent in homosexual couples (87.3%). About half of respondents acknowledged that direct contact is the most prevalent transmission mode (56.3%). However, few knew that the monkeypox virus could also be transmitted via blood and during pregnancy (36.5%). Surprisingly, only 41.5% of respondents concurred that avoiding contact with an infected person is one of the keys to preventing the spreading of the monkeypox virus. According to the question regarding the severity of clinical features, most participants (n = 193; 61.5%) agreed that the monkeypox virus causes a mild disease, and approximately 63.1% knew the appearance of the most common symptoms. Unfortunately, most respondents (75.2%) were unaware of the incubation period, whereas 74.5% were aware that blood samples are one of the most important tests for confirming a diagnosis of monkeypox virus.

Regarding licensed vaccines, approximately 73.6% of respondents correctly identified the absence of monkeypox virus vaccines. A large proportion of respondents (78.4%) believed that Saudi Arabia is at risk of being affected. In contrast, a smaller proportion acknowledged that the country is currently afflicted with chicken pox, a disease that resembles the monkeypox virus (37.2%). Nearly the same number of participants answered correctly (48.2%) and incorrectly (51.9%) that fluid therapy is the most common supportive treatment for this disease.

**Table 2:** Responses of medical students to the knowledge questions about the monkeypox virus (N=314)

Statement	Correct	Wrong
	N (%)	N (%)
The first time the monkeypox virus was discovered (isolated) in 1958	20 (6.4)	294 (93.6)
The first place the monkeypox virus was discovered (isolated) in Africa	62 (19.7)	252 (80)
Currently, the most affected area by the monkeypox virus is Africa	148 (47.2)	166 (52.9)
Currently, the monkeypox virus has become a global epidemic	294 (93.8)	20 (6.4)
The Monkeypox virus disease is re-emerging disease	117 (37.3)	197 (62.7)
The most common method of the monkeypox virus transmission is skin contact	177 (56.3)	137 (43.6)
The Monkeypox virus can be transmitted vertically from mother to child	115 (36.5)	199 (63.4)
Blood-borne transmission of the MPXV is possible	84 (26.9)	230 (73.2)
The Monkeypox virus cannot be spread through food	120 (38.2)	194 (61.8)
The Monkeypox virus cannot be spread through the air	137 (43.6)	177 (56.4)
The Monkeypox virus is a mild disease in general	193 (61.5)	121 (38.5)
The most common symptoms of the monkeypox virus (fever, rash, swollen lymph nodes)	198 (63.1)	116 (36.9)
The typical incubation period of the monkeypox virus (5 - 21 days)	78 (24.9)	236 (75.2)
A blood sample is used to confirm the diagnosis of the monkeypox virus	234 (74.5)	80 (25.5)
The most important method for preventing the spread of the monkeypox virus disease in the communities is to avoid contact with infected individuals	130 (41.5)	184 (58.6)
There was a licensed the monkeypox virus vaccine available at the time of this study	231 (73.6)	83 (26.4)
The most common the monkeypox virus treatment is supportive therapy e.g., fluid	151 (48.2)	163 (51.9)
Saudi Arabia is affected by a disease that resembles the monkeypox virus which is chickenpox virus	117 (37.2)	197 (62.7)
Monkeypox virus can be imported to Saudi Arabia	246 (78.4)	68 (21.7)
Monkeypox virus outbreaks in 2022 were noted to be related to homosexuality	274 (87.3)	40 (12.7)

Table 3 displays the relationship between socioeconomic variables and monkeypox virus knowledge level. The classification of monkeypox virus knowledge scores among medical students revealed that 72% had poor knowledge levels while 28% had good knowledge levels. There was a significant association (p<0.01) between monkeypox virus knowledge level and the age of participants, with 69% of those over the age of 21 having inadequate monkeypox virus knowledge. In addition, a significant correlation (p<0.001) was observed between monkeypox virus knowledge level and educational year of study participants, with the percentage of those with a good knowledge level increasing as the educational year progressed, except for those in the fifth year (27.7%) who obtained a lower score than those in the fourth year (33.5%). There is also a statistically significant difference (p<0.01) in GPA, with 35.3% of those with a GPA ≥ 3.75 achieving a good level of knowledge. Interestingly, a statistically significant difference (p<0.05) was observed between the education level of participants' fathers and monkeypox virus knowledge level, indicating that a poor monkeypox virus knowledge level increased as participants' fathers' education level increased. Participants who were not infected with COVID-19 had a significantly lower monkeypox virus knowledge level (89.5%) than those who were infected (p<0.001). Those who did not participate in monkeypox virus training programs had significantly lower monkeypox virus knowledge (92.3%) than those who did (p<0.001), indicating a statistically significant difference.

**Table 3:** Relationship between sociodemographic characteristics and the level of knowledge about the monkeypox virus among study participants (N=314) * Significant P-value < 0.05

Variables	Knowledge level		X^2^	P
	Poor (n =226)	Good (n =88)		
	N (%)	N (%)		
Gender				
Male	91 (69.4)	40 (30.6)	0. 70	0.402
Female	135 (73.8)	48 (26.2)		
Age				
≤21	42 (89.4)	5 (10.6)	8.28	0.003*
>21	184 (69)	83 (31)		
Educational year 2nd year	61 (91)	6 (9)	28.09	< 0.001*
3rd year	39 (81.2)	9 (18.8)		
4th year	42 (65.5)	22 (33.5)		
5th year	47 (72.3)	18 (27.7)		
6th year	37 (52.8)	33 (47.2)		
GPA (out of 5)				
<2.5	79 (85.9)	13 (14.1)	12.7	0.002*
2.5 – 3.74	64 (68.1)	30 (31.1)		
≥ 3.75	83 (64.8)	45 (35.2)		
Father's education				
< University level	62 (83.7)	14 (16.3)	4.58	0.032*
≥University level	164 (74.5)	74 (25.5)		
Mother's education				
< University level	57 (79.1)	15 (20.9)	2.39	0.121
≥University level	169 (69.8)	73 (30.2)		
Infected with COVID-19				
Yes	106 (58.9)	74 (41.1)	35.80	< 0.001*
No	120 (89.5)	14 (10.5)		
Vaccinated against COVID-19				
Yes	206 (72.3)	79 (27.7)	0.14	0.704
No	20 (69)	9 (31)		
Received training program about MPXV				
Yes	47 (39.2)	73 (60.8)	103.64	< 0.001*
No	179 (92.3)	15 (7.7)		

The attitudes of medical students regarding the monkeypox virus, emerging diseases, and travel medicine are presented in Table 4. Nearly half of the respondents agreed that the monkeypox virus could be transmitted to KSA (45.9%), that the global population would be able to control the monkeypox virus epidemic (45.2%), and that adequate monkeypox virus prevention and control measures are available (48.4%). 51.2% disagreed with negative feelings about the monkeypox virus, and 49.1% disagreed that traveling to monkeypox virus-infected countries is dangerous. About 61.4% agreed that media coverage of the monkeypox virus could affect its global prevention and that travel medicine should be a required course during my medical schooling (61.8%). 64% of respondents disagreed that the monkeypox virus will become a new pandemic, and its impact will be similar to COVD-19. The majority of respondents (71%) expressed a desire to learn more about the epidemiology of emerging diseases.

**Table 4:** Attitudes about monkeypox virus, emerging illnesses, and travel medicine among study participants (N=314)

Sentence	Strongly agree	Agree	Neutral	Disagree	Strongly disagree
	No (%)	No (%)	No (%)	No (%)	No (%)
I am sure that the global population will be able to control the monkeypox virus epidemic	75 (23.9)	67 (21.3)	89 (28.4)	48 (15.3)	35 (11.1)
I believe that monkeypox virus prevention and control measures are adequately available	64 (20.4)	88 (28)	110 (35)	33 (10.5)	19 (6.05)
I have negative feelings about the monkeypox virus	28 (9)	41 (13)	84 (26.8)	94 (30)	67 (21.2)
I believe that monkeypox virus adds additional strain on the healthcare systems of the affected countries	24 (7.6)	37 (11.8)	140 (44.6)	52 (16.6)	61 (19.4)
I think that monkeypox virus can be transmitted to KSA	48 (15.3)	96 (30.6)	82 (26.1)	38 (12.1)	50 (15.9)
I believe that media coverage of monkeypox virus may have an impact on its global prevention	95 (30.2)	98 (31.2)	52 (16.6)	41 (13)	28 (9)
I think monkeypox virus will become a new pandemic, and its impact will be like COVD-19	36 (11.5)	24 (7.6)	53 (16.9)	120 (38.2)	81 (25.8)
I would like to learn more about the epidemiology of new emerging diseases	140 (44.6)	83 (26.4)	61 (19.4)	19 (6)	11 (3.5)
I believe that travel medicine should be a required course during my medical school education	79 (25.2)	115 (36.6)	82 (26.1)	28 (8.9)	10 (3.2)
I believe that traveling to monkeypox virus infected countries is risky	47 (15)	51 (16.2)	62 (19.7)	72 (22.9)	82 (26.2)

## Discussion

The clinical management of monkeypox virus outbreaks requires the close collaboration of knowledgeable healthcare professionals. An adequate understanding of the disease is required for all aspects of disease management, including case detection, immunization, and the provision of ultimate medical care. Therefore, this study aimed to investigate medical undergraduates' knowledge, awareness, and attitudes regarding the monkeypox virus. According to our knowledge, this is the first study to assess students' knowledge and attitudes regarding the potential spread of the monkeypox virus. According to the data presented in our study, most respondents were aware of the monkeypox virus epidemic. Regarding the mode of transmission, more than half of the respondents believed it was a sexually transmitted disease. In contrast, they were also aware of blood-borne transmission and maternal-fetal transmission. Some students were also unlikely to be aware of the commonly exhibited symptoms and incubation period. Less than half of the respondents were aware of fluid therapy, despite it being the most common form of supportive therapy. 72% of respondents had poor levels of knowledge, compared to 28% who had adequate levels. These results were comparable to those of a study conducted by Ibrahim et al. [23] to determine 426 medical students' knowledge of the Zika virus. They discovered that 77.5% of their parents had inadequate knowledge of the Zika virus. In both studies, the lack of disease emergence in Saudi Arabia during data collection may have contributed to these results. The Zika virus was confined primarily to America, whereas the monkeypox virus is prevalent in African nations [23].

Harapan et al. [18] conducted a similar study in Indonesia to determine general practitioners' knowledge about the monkeypox virus. They included 432 general practitioners as participants in their study. The vast majority of individuals (96.6%) know that monkeypox is caused by a virus that may also cause smallpox. Fewer practitioners correctly answered other questions concerning the mode of transmission, the spread of the disease as an epidemic, and the management of the disease. The absence of the disease in Indonesia was cited as the reason for general practitioners' lack of knowledge. Most participants in the Indonesian study (73.6%) obtained their information from online media [18]. This is comparable to the current study, which found Twitter to be the most popular information source. Studies suggest that a lack of guided sources may lead to knowledge conflicts among students [24]. In addition, Harapan et al. [18] observed that practitioners who graduated from universities located in more developed regions possessed a higher level of knowledge. Current research revealed a strong correlation between student's knowledge and their fathers' level of education. Along with the findings of Alshahrani et al. [17], those who had participated in the training program for the monkeypox virus demonstrated a significantly higher level of knowledge than those who had not.

According to studies, training in healthcare facilities boosts medical practitioners' confidence [25,26]. Approximately half of the students exhibited confidence in their ability to combat the epidemic using available prevention and control measures. This result was consistent with that of Harapan et al.'s study, they conducted a cross-sectional survey of primary care physicians to determine their confidence levels in dealing with the monkeypox virus and discovered comparable results [19]. Once students were exposed to information about the monkeypox virus throughout their medical training, their confidence in treating patients with the virus increased. A comparative analysis of knowledge levels regarding Ebola, MERS, and the Zika virus supports this conclusion. In Saudi Arabia, the higher prevalence of MERS was accompanied by a greater proportion of well-informed participants than in the other two diseases. These statistics highlight the necessity of incorporating courses on the epidemiology of emerging diseases into the undergraduate and graduate levels of medical education. An observational study conducted by Al-Thaqafy et al. found that knowledge scores on infectious disease in the Saudi Arabian community, as well as attitudes and practices, increased after the educational intervention was implemented in the form of educational leaflets, group discussions, visual shows, and lectures [27]. A positive correlation was observed between GPA and a high level of knowledge. Until the fourth year of college, students' knowledge improved significantly, whereas those in the fifth year received lower grades. Similarities were found between this finding and the Zika study [23], those who had previously contracted COVID-19 had a greater understanding of the monkeypox virus. In addition, certain findings were observed in COVID-vaccinated individuals. Students who had been infected were more knowledgeable about not only COVID-19 but also other emerging infectious diseases and their epidemiology. Sixty percent of respondents were not in agreement that the re-emergence of the monkeypox virus could take the form of a pandemic like COVID-19. Approximately 60% of them were unaware that human-to-human contact is an important mode of transmission and that avoiding it can significantly reduce the risk of transmission. Recent studies on outbreaks of monkeypox virus in African nations have shown that secondary attacks among unvaccinated contacts may occur at a rate as high as 10%, with a fatality rate of 6% [28,29].

Most general practitioners in Indonesia (98.2%) agreed that the government should provide health care workers with monkeypox virus training, and 88.8% of general practitioners in Indonesia recommended that the virus be included in Indonesia's National Medical Curriculum [19]. Similarly, most students in our study were curious about the epidemiology of new emerging diseases, monkeypox virus, and travel medicine. This finding necessitates the incorporation of these essential medical disciplines into the medical curriculum. The present study provides information on medical students' current knowledge and awareness regarding the newly emerging epidemic of the monkeypox virus. It highlights the need for the addition of new curriculum topics. It was observed that higher-semester students were significantly more aware of the monkeypox virus than those enrolled in lower semesters. This was anticipated and consistent with the findings of Albarrak et al. [30], who observed that senior Saudi medical students had significantly greater knowledge of the Swine Flu and H5N1 influenza viruses than their junior counterparts. Ibrahim et al. came to comparable conclusions. They reported that senior medical students in Saudi Arabia were significantly more knowledgeable about the Zika virus than their junior counterparts [23].

Our study revealed that both sexes possessed low levels of general knowledge regarding the monkeypox virus. In addition, it was observed that the respondents preferred self-administered questionnaires. However, our study's findings contradicted those of Albarrak et al. [30] they observed that male Saudi medical students were significantly more knowledgeable about the Swine Flu and H5N1 influenza viruses than their female counterparts. Ibrahim et al. [23] came to comparable conclusions reporting that male Saudi medical students were significantly more knowledgeable about the Zika virus than their female counterparts.

In addition, it was found that respondents preferred face-to-face interviews. Ibrahim et al. [23] came to comparable conclusions. According to their findings, respondents favored face-to-face interviews. A possible explanation for this disparity is that face-to-face interviews were deemed the most effective data collection method. Face-to-face interviews were deemed more effective than self-administered questionnaires because they allowed respondents to respond to the questions in detail and with full comprehension. They are also considered the most effective method for determining the respondents' attitudes and beliefs. However, there were disadvantages associated with face-to-face interviews. Interviews were time-consuming for both the interviewer and the interviewees. Many interviewers were needed to complete the study within the allotted time frame. However, these resources are not easily accessible in many locations. Face-to-face interviews were linked to the possibility of interviewer bias. This may affect the study's findings. In addition, these interviews were associated with the risk of social desirability and respondents may be hesitant to respond to certain questions. However, self-administered questionnaires were deemed more effective than face-to-face interviews because they allowed respondents to respond to questions in detail and with full comprehension. In addition, they were believed to be the most objective method for exploring the respondents' attitudes and beliefs.

However, this study was not free from limitations. First, because the study was cross-sectional in nature, no causal link between the dependent and independent variables could be established. Second, this study did not include the estimated sample size due to the exclusion procedures. Third, the study was conducted at only one institution; therefore, the findings cannot be generalized to the broad spectrum of the country. Regional differences in the facilities made available to students may account for possible selection bias in the study's findings. Fourth, the scales of knowledge and attitude test were not validated, as we did not perform any factor analysis to see the construct validity of the questionnaire. Lastly, cognitive and reporting bias was possible in the questionnaire, which was based on a subjective approach.

## Conclusions

In general, this study showed that medical students had inadequate knowledge and awareness about the monkeypox virus. Students’ age, GPA, fathers’ education level, and training received about the monkeypox virus were significantly associated with the level of knowledge about the monkeypox virus. Although they were optimistic about the system's ability to manage future outbreaks of the monkeypox virus in Saudi Arabia, they were unaware of the disease transmission and possessed a limited understanding of the virus. These findings emphasize the urgent need to increase their knowledge because controlling outbreaks requires significant cooperation from knowledgeable and skilled healthcare providers. Our research suggests that, in order to make it easier for students to learn about new epidemic outbreaks, such subjects be covered in educational courses, and public health training and awareness programs.

## Appendices

Survey of Medical students’ perception regarding the re-emerging monkeypox virus: A cross-sectional study from Saudi Arabia

استبيان لقياس مدى معرفة طلاب الطب بفيروس جدري القردة: دراسة مقطعية من المملكة العربية السعودية

Consent question: I am a medical student at King Khaled University, and I voluntarily agree to be a part of this research.

أنا طالب/ة طب في جامعة الملك خالد، وأوافق على المشاركة في تعبئة الاستبيان وأن أكون جزءًا من هذا البحث • Yesنعم • No    لا         

Gender:          الجنس: • Male   ذكر • Female انثى    

Age:  العمر: • ≤21         أكبر أو يساوي 21 • >21 أقل من 21              

Educational year: السنة الدراسية : • 2nd year  السنة الثانية • 3rd year السنة الثالثة • 4th year  السنة الرابعة • 5th year السنة الخامسة • 6th yearالسنة السادسة         

GPA (out of 5) المعدل التراكمي (من 5) • <2.5 • 2.5 - 3.74 • ≥3.75

Father’s education مستوى تعليم الأب • < University level <مستوى الجامعة • ≥ University level ≥مستوى الجامعة         

Mother’s education مستوى تعليم الأم • < University level<مستوى الجامعة • ≥ University level ≥مستوى الجامعة        
 

Infected with  COVID-19هل أصبت بفيروس كورونا - 19 • Yes نعم • No  لا         
 

Received training programs about MPXV   حصلت على دورات تدريبية حول موضوع فيروس جدري القردة • Yes   نعم • No    لا      
 

Source of Information about MPXVمصدر معلوماتك حول موضوع فيروس جدري القردة • Twitter  تويتر • Snapchat السناب شات • Television التلفاز • WhatsApp group قروبات الوتس اب • Friends and relatives الأقارب و الأصدقاء • Research articlesمقالات بحثية                      

Knowledge Question about MPOX:

أسئلة المعرفة عن جدري القردة:

The first time MPXV was discovered (isolated) (1958(

اول مرة تم اكتشاف فيروس جدري القردة (1958) • Yes نعم • Noلا       
 

The first place MPXV was discovered (isolated) in (Africa)

أول مكان تم اكتشاف فيه فيروس جدري القردة (أفريقيا) • Yes نعم • Noلا        
 

Currently, the most affected area by MPXV (Africa)

حاليا, المنطقة الأكثر تضررا بفيروس جدري القردة هي افريقيا • Yesنعم • Noلا        
 

Nowadays MPXV started to be epidemic all over the world (Yes)

في الوقت الحاضر, بدا فيروس جدري القردة بالانتشار في جميع انحاء العالم (نعم) • Yesنعم • Noلا           
 

MPXV disease type (Re-emerging disease)

نوع مرض فيروس جدري القردة (عودة ظهور المرض) • Yesنعم • Noلا           
 

The most common method of MPXV transmission (Contact)

الطريقة الأكثر شيوعًا لنقل فيروس جدري القردة (الاتصال الجسدي او الملامسة) • Yesنعم • Noلا      
 

MPXV can be transmitted vertically from mother to child

يمكن انتقال فيروس جدري القردة عن طريق المشيمة من الأم للطفل • Yes نعم • Noلا      

Blood-borne transmission of the MPXV is possible

من الممكن انتقال فيروس جدري القردة عن طريق الدم • Yesنعم • Noلا          
 

MPXV cannot be spread through food

لا يمكن ان ينتشر فيروس جدري القردة من خلال الأكل • Yesنعم • Noلا          
 

MPXV cannot be spread through air

لا يمكن ان ينتشر فيروس جدري القردة من خلال الهواء • Yesنعم • Noلا        
 

MPXV is a mild disease in general

فيروس جدري القردة يعتبر مرض خفيف بشكل عام • Yesنعم • Noلا         
 

The most common symptoms of MPXV (fever, rash, swollen lymph nodes)

أكثر أعراض فيروس جدري القردة الأكثر شيوعا (حمى, طفح جلدي, تورم الغدد الليمفاوية) • Yes نعم • Noلا      
 

The typical incubation period of MPXV (5 - 21 days)

فترة الحضانة النموذجية لفيروس جدري القردة هي (5 -21 يوم) • Yesنعم • Noلا      
 

A blood sample is used to confirm the diagnosis of MPXV

تستخدم عينة الدم لتأكيد تشخيص فيروس جدري القردة • Yesنعم • Noلا       
 

The most important method for preventing the spread of MPXV disease in the communities is (Avoid contact with infected individuals)

أهم طريقة لمنع انتشار مرض فيروس جدري القردة  في المجتمعات هي (تجنب الاتصال و المخالطة مع الأفراد المصابين) • Yesنعم • Noلا        
 

There was a licensed MPXV vaccine available at the time of this Study

كان هناك لقاح لفيروس جدري القرود مرخص و متاح في وقت هذه الدراسة • Yesنعم • Noلا        
 

The most common MPXV treatment (supportive therapy e.g.,fluid)

علاج مرض فيروس جدري القرود الأكثر شيوعا هو (العلاج الداعم مثل السوائل) • Yesنعم • Noلا      

Saudi Arabia is affected by a disease that resembles MPXV (chickenpox virus)

في المملكة العربية السعودية يوجد مرض شبيه فيروس جدري القردة و هو (فيروس جدري الماء) • Yesنعم • Noلا     
 

MPXV can be imported to Saudi Arabia (Yes)

يمكن انتقال فيروس جدري القردة الى المملكة العربية السعودية • Yesنعم • Noلا     
 

MPXV outbreaks in 2022 were noted to be related to homosexuality

لوحظت تفشيات فيروس جدري القردة في عام 2022 بأنه مرتبط بالمثلية الجنسية • Yesنعم • Noلا     
 

I'm sure that the global population will be able to control the MPXV epidemic

أنا متأكد من أن سكان العالم سيكونون قادرين على السيطرة على وباء فيروس جدري القردة • Strongly agreeاوافق بشدة • Agree أوافق • Neutralمحايد • Disagreeلا أوافق • Strongly disagreeلا أوافق بشدة    
 

I believe that MPXV prevention and control measures are adequately available

اعتقد ان تدابير الوقاية و السيطرة على فيروس جدوي القردة متوفرة بشكل كاف 

• Strongly agreeاوافق بشدة • Agree أوافق • Neutralمحايد • Disagreeلا أوافق • Strongly disagreeلا أوافق بشدة I have negative feelings about the MPXV

لدي مشاعر سلبية تجاه فيروس جدري القردة • Strongly agreeاوافق بشدة • Agree أوافق • Neutralمحايد • Disagreeلا أوافق • Strongly disagreeلا أوافق بشدة I believe that MPXV adds additional strain on the healthcare systems of the affected countries

اعتقد ان فيروس جدري القردة يضيف ضغطا إضافيا على انظمة الرعاية الصحية في البلدان المتضررة • Strongly agreeاوافق بشدة • Agree أوافق • Neutralمحايد • Disagreeلا أوافق • Strongly disagreeلا أوافق بشدة I think that MPXV can be transmitted to KSA

اعتقد ان فيروس جدري القردة يمكن انتقاله الى المملكة العربية السعودية • Strongly agreeاوافق بشدة • Agree أوافق • Neutralمحايد • Disagreeلا أوافق • Strongly disagreeلا أوافق بشدة I believe that media coverage of MPXV may have an impact on its global prevention

اعتقد ان التغطية الإعلامية لفيروس جدري القردة قد يكون لها تأثير على الوقاية العالمية • Strongly agreeاوافق بشدة • Agree أوافق • Neutralمحايد • Disagreeلا أوافق • Strongly disagreeلا أوافق بشدة    
 

I think MPXV will become a new pandemic, and its impact will be like COVD-19

اعتقد أن فيروس جدري القردة سيصبح وباء جديدا وسيكون تاثيره مثل فيروس كورونا-19 • Strongly agreeاوافق بشدة • Agree أوافق • Neutralمحايد • Disagreeلا أوافق • Strongly disagreeلا أوافق بشدة    
 

I would like to learn more about the epidemiology of new emerging diseases

اود معرفة المزيد عن الأمراض المستحدثة في علم الأوبئة • Strongly agreeاوافق بشدة • Agree أوافق • Neutralمحايد • Disagreeلا أوافق • Strongly disagreeلا أوافق بشدة    
 

I believe that travel medicine should be a required course during my medical school education

اعتقد ان طب المسافرين يجب ان يكون مقررا مطلوبا اثناء فترة دراستي في كلية الطب • Strongly agreeاوافق بشدة • Agree أوافق • Neutralمحايد • Disagreeلا أوافق • Strongly disagreeلا أوافق بشدة    
 

I believe that traveling to MPXV-infected countries is risky

اعتقد ان السفر الى البلدان المصابة بفيروس جدري القردة محفوف بالخطر • Strongly agreeاوافق بشدة • Agree أوافق • Neutralمحايد • Disagreeلا أوافق • Strongly disagreeلا أوافق بشدة    
